# Plant Microbiome: An Ocean of Possibilities for Improving Disease Resistance in Plants

**DOI:** 10.3390/microorganisms11020392

**Published:** 2023-02-03

**Authors:** Sajad Ali, Anshika Tyagi, Hanhong Bae

**Affiliations:** Department of Biotechnology, Yeungnam University, Gyeongsan 38541, Gyeongbuk, Republic of Korea

**Keywords:** pathogens, immunity, microbiome, hormones, induced systemic resistance, SynComs

## Abstract

Plant diseases pose a serious threat to crop production and the agricultural economy across the globe. Currently, chemical pesticides are frequently employed to combat these infections, which cause environmental toxicity and the emergence of resistant pathogens. Moreover, the genetic manipulation of plant defense pathways and the breeding of resistant genes has attained limited success due to the rapid evolution of pathogen virulence and resistance, together with host range expansion. Additionally, due to climate change and global warming, the occurrence of multiple stresses during disease outbreak has further impacted overall crop growth and productivity, posing a serious threat to food security. In this regard, harnessing the plant beneficial microbiome and its products can provide novel avenues for disease resistance in addition to boosting agricultural output, soil fertility and environmental sustainability. In plant–beneficial microbiome interactions, induced systemic resistance (ISR) has emerged as a key mechanism by which a beneficial microbiome primes the entire plant system for better defense against a wide range of phytopathogens and pests. In this review, we provide the recent developments on the role of plant beneficial microbiomes in disease resistance. We also highlight knowledge gaps and discuss how the plant immune system distinguishes pathogens and beneficial microbiota. Furthermore, we provide an overview on how immune signature hormones, such as salicylic acid (SA), jasmonic acid (JA) and ethylene (ET), shape plant beneficial microbiome. We also discuss the importance of various high-throughput tools and their integration with synthetic biology to design tailored microbial communities for disease resistance. Finally, we conclude by highlighting important themes that need future attention in order to fill the knowledge gaps regarding the plant immune system and plant-beneficial-microbiome-mediated disease resistance.

## 1. Introduction

Plants are constantly challenged by different microbial pathogens that endanger their survival and pose a constant threat to global food security. In the past, numerous disease outbreaks have significantly impacted our agricultural output and economy, especially in countries with food shortages [1]. The Irish potato famine in the nineteenth century caused by *Phytophthora infestans*, which resulted in over two million fatalities and widespread migration from Ireland [2]. Similarly, *Cochliobolus miyabeanus*, causing brown spots on rice, was another significant plant disease and a disastrous epidemic that claimed the lives of over two million people. Food crops suffer significant yield losses worldwide due to microbial diseases and pests, with mean losses of 30.3% for rice, 22.6% for maize, 21.5% for wheat, 21.4% for soybeans and 17.2% for potatoes [1]. Plant diseases can reduce crop yields by 50% in some regions, primarily among smallholder farmers, leading to severe economic challenges [3]. Plant diseases also have a negative impact on species diversity, on downstream costs associated with control methods and on human health [3]. Emerging plant diseases and pest outbreaks have a major economic impact on agriculture, affecting food security, national security and human health [4]. In the future, it is anticipated that variations in the geographic distribution of pathogens in response to climate change and growing global commerce would make emerging plant diseases more prevalent and severe [5,6]. Recently, coffee rust outbreaks caused by *Hemileia vastatrix* in Central America have also caused huge yield losses and economic crises [7]. Unlike endemic diseases, which can be managed, emerging diseases can have severe consequences on contemporary agricultural and other input systems, necessitating prompt mitigation strategies. Currently, a worldwide epidemic is endangering the health of millions of people world-wide. Hence, the availability of nutritious and healthy food is critical to helping people escape poverty and achieving better health outcomes.

Over the last two decades, scientific breakthroughs have greatly benefited our efforts to manage plant infections. For example, genetically modifying plant immune components and breeding resistant genes are a few strategies that have been used to combat plant pathogens [8]. However, severe disease outbreaks are exacerbated by the rapid development of pathogen virulence and resistance, as well as host range expansion mainly under modern agricultural practices, which pose serious challenges to developing long-term disease-resistant cultivars. On the other hand, the application of chemical microbicides and fungicides has been partly successful, but their overuse has a negative impact on the environment, humans and the emergence of newly resistant pathogens [9]. For instance, a few examples of fungicide-resistant plant pathogens that represent a substantial danger to commercially significant crops include *Botrytis cinerea, Alternaria* sp., *Plasmopara viticola*, *Pseudocercospora fijiensis*, *Ramularia collo-cygni* and powdery mildew pathogens [10]. Pesticides can also have an impact on the beneficial microbiota, which normally protects plants from pathogens by competing with them, inhibiting their colonization, activating plant immunological pathways or secreting antimicrobial compounds [11]. At present, the rise of multi-pesticide-resistant pathovars is one of the most serious negative consequences of pesticide overuse in modern agriculture. Due to the limitations of current mitigation strategies for controlling disease outbreaks, there is a need to search for another alternative that is more effective and eco-friendly. In this context, harnessing the potential of plant beneficial microbiomes and their products is a viable strategy for the mitigation of plant diseases in sustainable agriculture, owing to their multifaceted functions that they provide to their hosts, such as promoting growth, providing nutrient availability, increasing soil fertility, being ecofriendly and boosting multiple stress resilience. Here, we focus on the recent developments on the role of plant beneficial microbiomes and disease resistance. We also discuss how plant immune signatures shape plant beneficial microbiomes. Furthermore, we provide an overview on how plant defense systems interact with beneficial and pathogenic microbes. Recent studies have shown that individual- and community-level features of plant microbiomes boost plant immunity in different crop systems. 

## 2. Plant Microbiome and Disease Resistance: A New Sustainable Approach for Controlling Emerging Disease Outbreaks

Plants in nature coexist with diverse microbial communities that can be beneficial, commensal and pathogenic. Plants interact with these diverse microbes in three key regions: the phyllopshere, endosphere and rhizosphere. The plant microbiome comprises a variety of elegant species, including fungi, bacteria, protozoa, archaea and viruses. Plant microbiomes can be beneficial in a variety of ways, including protecting the plant from harmful infections, improving tolerance to a wide range of abiotic stresses, increasing growth, health and production, and giving plants a competitive advantage in response to climatic changes [12,13,14]. However, the complex and dynamic interactions between plants and microbiomes are greatly influenced by the host, microbe and environment, forming a trio complex that influences their overall outcome [14].

**Figure 1:**
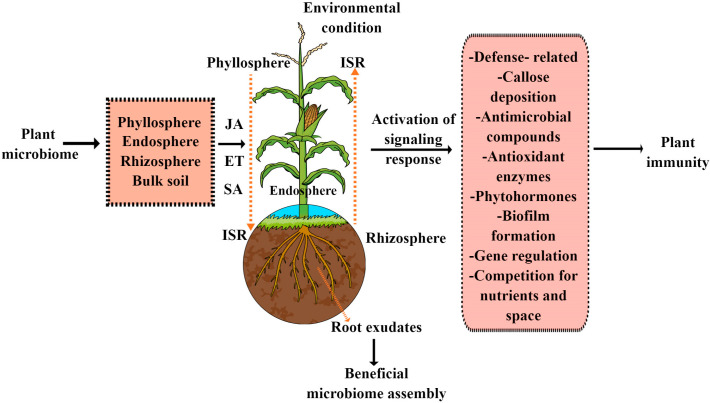
How plant microbiome (endosphere, phyllosphere, rhizosphere and bulk soil) confers disease resistance in plants. There are two types of microbiome-mediated disease resistance: direct, in which they secrete various antimicrobial chemicals, compete for resources and space and undergo callose deposition; and indirect, in which they activate the plant immune system, including ISR.

The plant microbiome plays a multifaceted role in protecting plants from pathogen attacks using different strategies, such as activating immune responses, induced systemic resistance (ISR) and callose deposition. They are also responsible for the production and excretion of the following: antimicrobial compounds, such as 2,4-diacetylphloroglucinol, proteases, chitinases, bacteriocins and siderophores; lipopeptides, such as iturin A, bacillomycin D and mycosubtilin; and volatile compounds. Moreover, they also inhibit pathogens by competition for nutrients and space, as shown in Figure 1. Interestingly, in plant–beneficial microbiome interactions, ISR has emerged as a key mechanism by which the beneficial microbiome primes the entire plant system for better defense against a wide range of phytopathogens and pests [15]. ISR is a generic term used for the induced resistance mechanism stimulated by both chemical and biological inducers that shield nonexposed plant organs against subsequent attacks by pathogenic bacteria and herbivorous pests. Generally, ISR is regulated by a complex of interconnected signaling cascades in which plant hormones such as JA, ET and SA, as well as their crosstalk, play an important regulatory role. However, in the majority of plant–beneficial microbiome interactions, ISR is predominantly regulated by the JA and ET hormonal cascades. This has been further proved in both mutant JA/ET and wild Arabidopsis plants, in which beneficial bacteria, such as *Pseudomonas fluorescens* WCS417r–ISR, *Serratia marcescens*, *Pseudomonas protegens* CHA0 and *Pseudomonas fluorescens* Q2-87, and beneficial fungi, such as *Penicillium* sp. GP16-2 and *Trichoderma harzianum* T39, trigger ISR in a JA/ET-dependent manner [16,17,18,19]. However, there are a few reports that show that beneficial microbes also trigger ISR through an SA-dependent manner. For example, beneficial bacteria such as *Pseudomonas aeruginosa* 7NSK2 fails to trigger ISR in SA mutant tomato plants [20]. As a result, it would be interesting to investigate how plant beneficial microbiomes activate ISR, because plant microbiomes contain a variety of microflora that are adapted to various lifestyles. Therefore, more research is needed to determine how JA/ET and SA hormones, as well as their interactions, play a role in microbiome-mediated ISR activation. In plants, beneficial bacteria, pathogens and insects all share the ISR-regulated linked signaling pathways. Consequently, more research is required to pinpoint the key participants in both pathogen- and microbiome-triggered ISR, which can reveal whether they utilize similar or dissimilar signaling or gene networks.

Many studies have reported that plant microbiota confers disease resistance against different pathogens of different lifestyles. For example, the activation of innate immunity in plants via root microbiota has been extensively characterized to provide resistance against numerous above-ground plant diseases *via* ISR [15,21]. ISR activation has been reported as an important driver of most plant-growth-promoting bacteria (PGPR)-mediated disease resistance in plants [15,22]. Previous studies have shown that that bacterial and oomycete infections in Arabidopsis leaves influence the root exudates to promote the assemblage of beneficial ISR triggering microorganisms [23,24]. In plants, root exudates play a major role not only by shaping the beneficial microbiome but also by providing disease resistance by directly inhibiting pathogen growth or activating the plant immune system. Plants secrete diverse root exudates, such as organic acids, vitamins, flavonoids, polysaccharides, amino acids and sugars that directly or indirectly confers host disease resistance. Many beneficial bacteria actively respond to root exudates by adjusting their transcriptional program toward traits involved in root colonization, activating ISR, chemotaxis, biofilm formation and energy metabolism which are important for inhibiting pathogens and disease progression. Although the role of root exudates in shaping the plant microbiome is well addressed, how they trigger the immune system is largely unknown. A growing body of studies on *Triticum avesticum* [25,26], *Arabidopsis thaliana* and *Beta vulgaris* [27] has revealed that pathogen-infected plants’ roots can attract helpful bacteria to rescue or safeguard future generations. Endosphere and rhizosphere microbiome members have also been found to inhibit plant diseases, such as the damping-off and take-all [28,29,30]. Similarly, Kwak et al. [31] reported that rhizospheric microbiota in tomato plants enhance disease resistance against wilt disease caused by *Ralstonia solanacearum*. Mendes et al. [32] also reported that rhizobacteria from the Pseudomonadaceae, Bacillaceae, Solibacteraceae and Cytophagaceae families are more prevalent in the rhizosphere of Fusarium-resistant bean cultivars. Similarly, another study revealed the role of the beneficial microbiome in mitigating the negative effects of potato scab disease, identifying key genera such as Geobacillus and Curtobacterium [33]. Numerous studies have shown that the beneficial microbiome directly suppresses pathogens through a variety of strategies, including the synthesis of antimicrobial compounds, hyperparasitism and competition for nutrients and space [34,35]. All of these strategies result in pathogen limitation but may vary from microbe to microbe. For example, Fusarium-vulnerable cucumber plants often assemble helpful microorganisms, such as Comamonadaceae and Xanthomonadaceae, to inhibit the Fusarium pathogen by producing higher levels of organic acids [36]. A previous study reported that *Pseudomonas fluorescens* WCS417 promotes callose deposition at the pathogen entry site and also triggers the expression of defense signature genes (pathogenesis-related genes) that restrict pathogen entry and disease progression [37]. Recently, a seed-endophytic *Sphingomonas melonis* strain was reported to provide disease resistance against *Burkholderia plantarii*, which causes seedling blight in rice [38]. Arbuscular mycorrhiza fungi (AMF) have also been shown in numerous studies to increase plant resilience to a variety of diseases. For example, mycorrhiza colonization in different crop plants provides disease resistance against numerous phytopthogens, such as *Pyrenochaeta terrestris*, *Fusarium oxysporum* f. sp. Lycopersici, *Phytophthora nicotianae* var. parasitica, *P. parasitica* and *Pseudomonas syringae* [39,40,41]. Additionally, AMF activate ISR and systemic acquired resistance (SAR), which provides disease resistance to pathogens from various lifestyles. Deciphering how AMF and a beneficial microbiome work together can therefore provide novel perspectives on plant disease resistance in sustainable agriculture. Furthermore, we summarize the role of plant beneficial microbiomes and their products in disease resistance in Table 1. 

**Figure 2:**
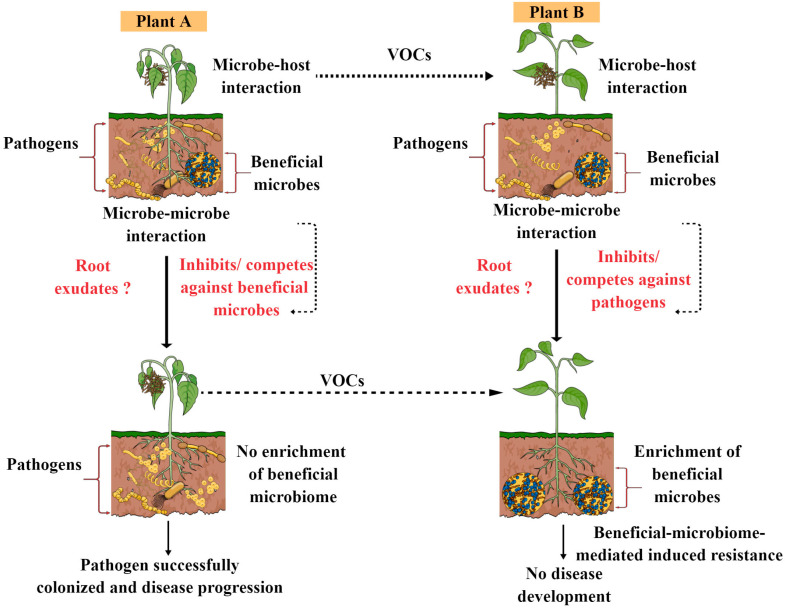
Schematic diagram showing how pathogen attack their hosts, either suppressing or enriching the beneficial microbiome and in turn leading to either disease resistance or disease progression. Susceptible plants may release VOCs, which can trigger immune activation and plant root exudation and can influence beneficial microbiome assembly, ultimately leading to disease resistance.

Recently, a new hypothesis has emerged in plants in the context of stress and plant beneficial microbiome recruitment, known as the ‘cry for help’. According to this cry for help hypothesis, plants actively recruit or enrich specific microorganisms during stress conditions that can protect them from detrimental effects and that can provide an array of growth-promoting benefits. This hypothesis was further studied under diverse stressful conditions in different plants, and the results were astounding, demonstrating how plants recruit their beneficial microbiomes to fend off pathogen attacks and gain further growth benefits from them [26]. In this review, we made one model showing how pathogen attacks in their hosts either suppress or enrich the beneficial microbiome, which in turn leading to either disease resistance or disease progression, as shown in Figure 2. However, there are many factors that govern the overall success or failure of plant beneficial microbiome and pathogen battles. For instance, the lifestyle of plant pathogens, plant species, environmental factors, root exudate chemistry, the plant immune system and signatures can have a significant effect (both individually and in combination) on plant beneficial microbiome and pathogen interactions. We are still in the early stages of understanding the complexity of plant beneficial microbiome and host disease resistance. Hence, future research should concentrate on determining how the plant beneficial microbiome influences disease resistance in both susceptible and resistant cultivars and how it varies between the two, which can provide novel opportunities to develop disease-resistant cultivars and control future disease outbreaks. Moreover, it would be interesting to explore how susceptible plants releases volatile compounds (VOCs) during pathogen attacks or disease development that can trigger the plant immune system or microbiome assembly in neighboring plants, making them alert and resistant to desired pathogen attacks. VOCs function as airborne signals in plant–plant or plant–microbe communication, which can assist plants in developing and coping with stress in both direct and indirect ways. It is well documented that plants release a wide range of volatile organic molecules when they become infected by pathogenic microorganisms, such as aromatics, terpenes, fatty acid derivatives and nitrogen-containing compounds, as well as volatile phytohormones, methyl jasmonate and methyl salicylate, which seem to provide disease resistance either directly or indirectly [51]. In the future, the integration of analytical and molecular tools is required to decipher the role of VOCs produced by plants or the beneficial microbiome in disease resistance in different crop pathology systems. Moreover, how VOCs from plants and beneficial microbiomes share or differ in triggering the plant immune system is one of the most exciting research area in the field of plant microbiome.

## 3. Surveillance of Pathogenic and Beneficial Microbes by the Plant Immune System

**Figure 3:**
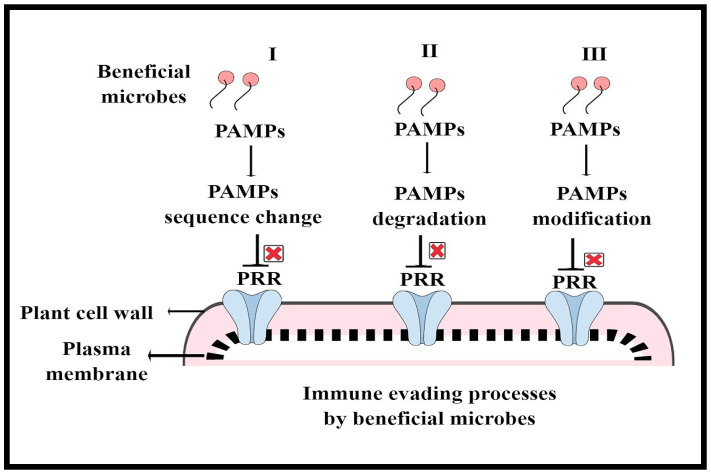
How beneficial microbes evade the plant immune system: (I) PAMPs sequence change; (II) PAMPs degradation; (III) PAMPs modification.

Plants, being sessile, constantly interact with a diverse group of microbes (beneficial and pathogenic) of different lifestyles, which either supports or limits their growth and development. However, plants have sophisticated immune systems that recognize these invaders and respond quickly to inhibit pathogen entry or disease progression [52,53,54]. To counteract various biotic stressors, plants employ both preformed (structural and bio-chemical) and inducible defense mechanisms. In other words, to detect and react to pathogen infections, plants primarily depend on two tier levels of their innate immune system, namely pattern recognition receptors (PRRs) on the cell surface to recognize microbe-associated molecular patterns (MAMPs) or host-derived damage-associated molecular patterns (DAMPs), also called PAMP-triggered immunity (PTI) and disease resistance (R) proteins that respond to effector molecules, also called effector triggered immunity (ETI) [43,55,56]. Various receptor-like kinases (RLKs) and receptor-like proteins (RLPs) have been discovered to serve as PRRs [46]. Interestingly, RLKs and RLPs have become the hallmarks of plant stress biology, owing to their multifaceted sensing ability and their ability to modulate diverse developmental and adaptive responses. In plants, PTI is established through transcriptional reprogramming caused by the activation of calcium-dependent protein kinases (CDPKs) and mitogen-activated protein kinase (MAPK) cascades by PRR complexes [57,58]. ETI typically causes a hypersensitivity response (HR), or programmed cell death, at the infection site and activates systemic defense signals, which prevent the spread of pathogens locally [59]. However, occasionally, pathogens evade the immune barriers, leading to detrimental outcomes in plants. For example, pathogens use effector molecules to interfere with PTI and host physiology for colonization and disease development. In recent years, numerous model and crop plants have been used to conduct extensive research on the interplay of molecular plant–pathogen interactions and the function of the plant immune system, which has led to the discovery of various key player receptors, effectors and immune signatures. Moreover, the above models, such as PTI and ETI, are based on how plants interact with pathogenic bacteria, despite the fact that they give a solid summary of the core ideas driving plant immunity. However, how beneficial microbes evade the plant immune system and how they are recognized and responded to by the plant immune system at the molecular level remain largely unknown. Previously, researchers have explored plant–pathogen interactions via the lens of an individual plant–microbe relationship, ignoring the plant beneficial microbiome, which has recently been recognized as the primary driver of plant–pathogen interaction outcomes [28,34,60]. Additionally, the plant immune system actively contributes to the building of the beneficial microbiome and modulates microbial homeostasis during environmental variation. Microbes use different strategies to evade or suppress PTI, such as (I) MAMP divergence, (II) MAMP degradation/sequestration and (III) MAMP modification, which involve an array of processes regulated by both microbes and plants. In the case of MAMP divergence, microorganisms may develop MAMP variations by modifying their sequence and structure that do not bind to or activate the matching plant PRR in order to avoid MTI. Second, during the process of MAMP degradation or sequestration, microorganisms can secrete a wide range of enzymes, such as hydrolases and proteases, to degrade their MAMPs or proteins that sequester MAMPs to keep them hidden from plant receptors. Microbes can also alter their MAMPs, such as flagellin, chitin and other molecules, that PRRS cannot identify in order to evade MTI [61,62]. There are many hypotheses that highlight how the beneficial microbiome may involve similar traits in order to evade PTI [63]. For example, it is possible that the plant microbiome may have developed identical MAMP modification techniques to avoid MTI, because flg22 and chitin are both widely present in plant microbiomes. Additionally, how beneficial bacteria alter host immune signaling components by secreting proteins and effectors that can help them invade plant immunity barriers is still largely unexplored. Therefore, future studies are required to pinpoint how the beneficial microbiome evades or modifies plant defense barriers, which can provide now insight on the plant microbiome and disease resistance. Furthermore, we summarize how the beneficial microbiome evades plant immunity in Figure 3. Moreover, deciphering how the immune system influences the plant beneficial microbiome is the most fascinating topic of research in plant stress biology. This can offer new insights for enhancing crop resilience to infections.

## 4. Role of Plant Defense Signatures in Shaping the Plant Beneficial Microbiome

**Figure 4:**
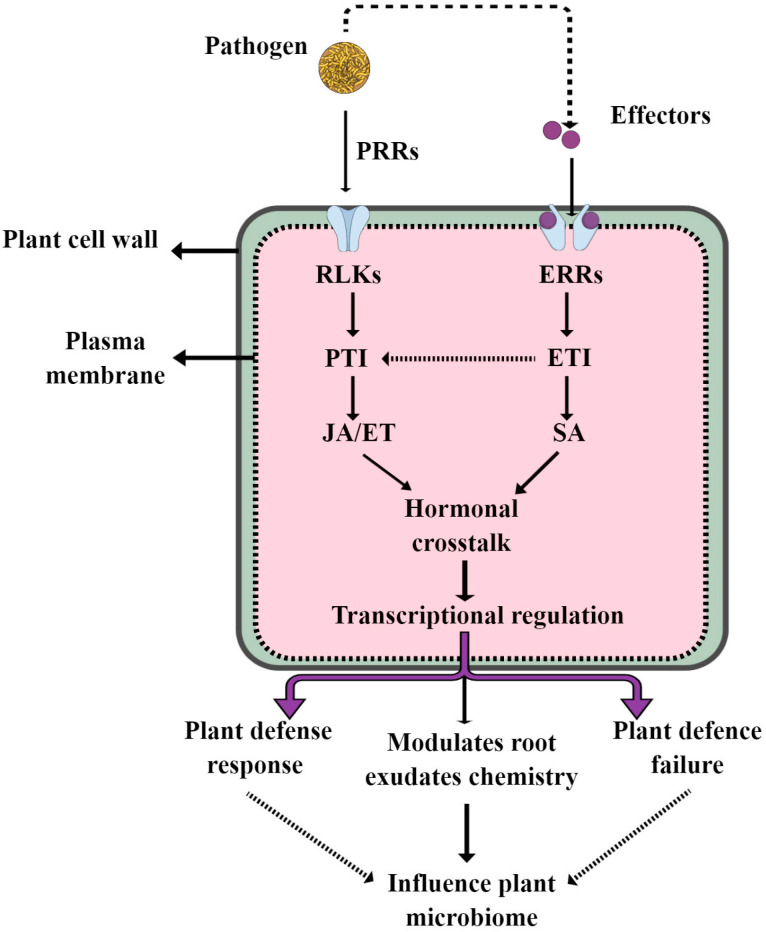
This model highlights how plant defense signatures such as SA, JA and ET influence the plant microbiome during pathogen interactions.

Plant beneficial microbiome assembly is dynamically controlled by complex interactions among hosts, microorganisms and environmental variables [14,64]. There are many excellent reviews covering how root exudates and environmental factors shape the plant beneficial microbiome under different conditions [13,14,65]. In this review, we focus on how plant defense signatures influence the plant beneficial microbiome. The plant immune system is a complex system regulated by different defense signature hormones, such as salicylic acid (SA), jasmonic acid (JA) and ethylene (ET). The role of these versatile hormones in plant defense responses is well understood in both model and crop plants. For example, SA-dependent defenses provide resistance to biotrophic pathogens, whereas JA- and ET-dependent defenses are effective against necrotrophic pathogens and herbivorous insects [66,67]. Recently, they have been identified as important drivers of plant beneficial microbiome assembly. For instance, Lebeis et al. [68] reported that SA modulates the colonization of the root microbiome via specific bacterial taxa in Arabidopsis. This study showed that SA knock-out Arabidopsis mutants have root microbiomes that differ from the wild-type relative abundance of specific bacterial families. On the other hand, NPR1 (SA receptor) mutants have reduced endosphere microbiome diversity, mainly alpha diversity, and also less endophyte colonization [69,70]. The JA pathway was also identified as an important driver of plant-immune-system-mediated microbiome assembly in Arabidopsis. This study showed that the mutant JA pathway in Arabidopsis plants, namely myc2 and med25, have distinct microbial communities when compared to wild-type plants [71]. Similarly, in Arabidopsis, exogenous treatment with JA was proven to boost Arabidopsis rhizosphere alpha diversity while also enriching many important beneficial microbial taxa [72]. ET, which often acts synergistically with JA in defense signaling, also influences beneficial microbiome assembly. For example, in peanuts, exogenous ET increases rhizosphere alpha diversity, particularly the amount of actinobacteria, while decreasing the abundance of acidobacteria [70]. However, the effect of plant defense signatures on plant beneficial microbiome assembly varies among plant species and compartments. For example, it has been shown that JA plays a different role in epiphytic Arabidopsis leaf communities and wheat (*T. aestivum*) root endosphere community composition [73,74]. These findings imply that the effect of plant hormones on the root microbiota may vary by species. Understanding how plant hormones affect the root beneficial microbiome in crops is crucial for manipulating plant–microbiome interactions for better plant productivity. However, many questions remain unanswered: (1) How do plant defense hormones affect root exudates, which in turn influence the beneficial microbiome? (2) How do trio SA/JA and ET crosstalk influence the plant beneficial microbiome during pathogen infections? (3) How do plant defense signatures interact with other drivers of microbiome assembly, and what is their effect on the beneficial microbiome? A deeper understanding into the manipulation of the plant microbiome by the endogenous pathway may provide novel breeding and engineering strategies to improve sustainable yields and crop resilience. Furthermore, we summarize how plant defense signatures influence plant beneficial microbiome assembly in Figure 4.

## 5. Developing Disease-Resilient Microbial Communities for Disease Resistance

**Figure 5:**
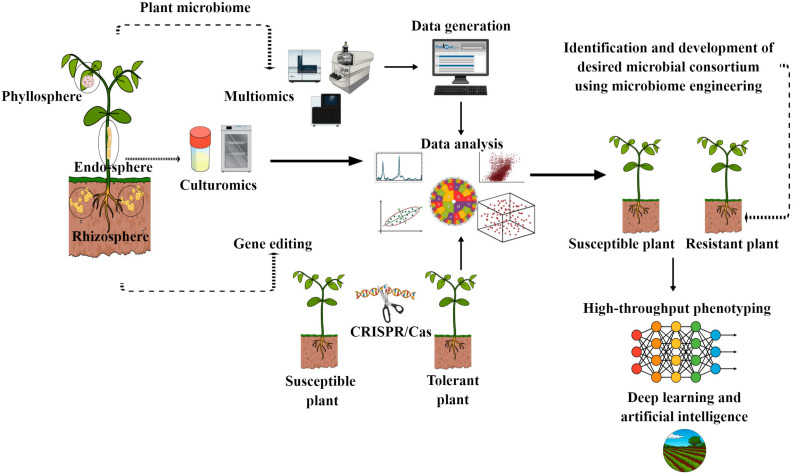
Overview of different molecular, synthetic and high-throughput tools for developing tailored beneficial microbiome for disease resistance in sustainable agriculture. This figure also shows the role of phenotyping, artificial intelligence and deep learning for monitoring the functionaries of tailored beneficial microbiomes under field conditions.

Over the last decade, our understanding of the plant beneficial microbiome has grown dramatically. Integrated techniques, such as various multi-omics and microbiome engineering strategies, have significantly improved our understanding of the organization and dynamics of the plant microbiome and their interactions [13,14,75]. For instance, in wild and benzoxazinoid precursor mutant maize plants, a combined metagenomic and metabolomic analysis revealed that benzoxazinoid metabolites play an important role in the formation of the rhizosphere microbiome [76]. Similarly, Stringlis et al. [77] discovered that coumarin exudation from roots can influence the rhizosphere microbiome in Arabidopsis (wild and mutant) plants using combined metabolomics and shotgun metagenomics methods. A recent work used metagenomics and metabolomics to explore the effect of root-exuded triterpenes on root microbiota composition [78]. However, there are few studies on the plant beneficial microbiome and disease resistance. Nevertheless, plant beneficial microbiomes and their products are attracting increasing interest as a means of combating disease outbreaks under climatic changes due to their all-around performance against multiple stressors and their plant-growth-promotion traits. However, due to limited knowledge, many things remain unknown. In this context, the integration of multiomics can provide novel insights on how the plant immune system regulates plant beneficial microbiome assembly, root exudates chemistry and their selection. Moreover, the application of multiomics can help unravel how SA/JA and ET trigger transcriptional, metabolic and proteomic reprogramming, which influence plant beneficial microbiome assembly and which can in turn can promote growth and disease resistance. So far, different microbial members have been identified to inhibit infections; however, their applicability in the field is limited due to their reliance on numerous host and environmental parameters. Moreover, microbes vary in terms of their physiology, metabolism and susceptibility to temperature and moisture. Consequently, the composition of the plant beneficial microbiome may be directly impacted by climate change. Microbial communities living on the surface of plants, such as the phyllosphere, are expected to be more directly impacted by climate change than those inside plant tissue ecosystems, which tend to experience more constant environmental circumstances [79]. Therefore, the absence of above-ground rescuers can increase the likelihood of a pathogen or disease spreading to the plants’ above-ground parts. In this context, beneficial microbiome engineering and host gene editing may aid in overcoming these constraints [80]. Moreover, engineered plants that secrete exudates that encourage particular advantageous plant–microbe interactions may be possible, which can confer disease resistance and plant growth promotion. Previous studies have revealed that native microbiota can rescue their hosts from emerging disease outbreaks [81]. Therefore, in order to fully utilize the potential of the native microbiota, microbiome engineering or host editing may be the most practical strategies for creating effective, customized microbial consortia that can be used to manage future disease outbreaks. Owing to the complexity of plant–microbiome interactions, there remain many hidden secrets that limit our understanding and their impact on each other. However, the advent of new technologies such as deep learning, artificial intelligence and high-throughput phenotypic platforms is providing incredible insights in the plant microbiome world, aiding scientist to better understand their intricacy and develop new models of relationships between plants and their beneficial microbiomes. Furthermore, in Figure 5, we summarize various tools that can be used to explore the new frontiers of the plant beneficial microbiome world with respect to disease resistance. Numerous national and international policy authorities have acknowledged that it is crucial to manipulate the plant–soil microbiome in order to boost plant productivity in the face of climate change [79,82]. In the future, tailored microbial communities will be the most viable sources to prevent disease outbreaks in sustainable agriculture. Moreover, future breeding strategies may be expanded by understanding how wild relatives may involve plant genes in beneficial microbiome construction under disease outbreaks, which can help in identifying traits that can be used for developing disease-resistant crops.

## 6. Conclusions and Future Perspectives

At present, our agriculture is facing a number of challenges, such as global climate change, abiotic and biotic stressors, soil infertility, water shortages and pollution, all of which have a significant impact on crop output and pose a serious threat to food security. Similar to this, plant pathogens and the disease outbreaks they cause have had a major impact on our agricultural system for decades, causing enormous food and economic catastrophes. Currently, both endemic and emerging plant diseases are spreading and intensifying due to the increased rate of global climate change, mutations and the evolution of new pathovars, pathogen spillover and transmission via world food trade networks, which have made it difficult to control them with currently available treatments. Therefore, it is necessary to find new remedial tools that provide an efficient and long-lasting way to increase disease resistance and crop productivity sustainably. In light of this, utilizing the potential of plant beneficial microbiomes and their products is one of the most adaptable ways to combat infections and disease outbreaks in our agriculture system. The increased interest in the plant beneficial microbiome clearly results from its significant potential to offer environmentally friendly solutions in plant disease protection and cutting-edge tools to promote sustainability in agroecosystems, contributing to a new Green Revolution that is safe for humans and the environment [83]. Over the last 10 years, our understanding of plant–microbe interactions and their effects on crop resilience and production has significantly advanced as a result of omics and other molecular tools. However, we are beginning to understand this dynamic and intricate relationship between the beneficial microbiome of the plant and the effects it has on plant fitness and productivity. Nevertheless, in the past many studies have shown that plants shape their beneficial microbiome under different stress conditions in order to protect themselves. These studies indeed open new frontiers in the plant beneficial microbiome world. Unraveling the beneficial microbiome’s potential for crop resilience and productivity is challenging due to the intricacy of plant microbiome dynamics and the reliance on external factors. Moreover, our understanding of the significance of the plant beneficial microbiome in terms of ecology and function remains restricted, despite the fact that analytical studies of plant–microorganism interactions have expanded in recent years. Plants have diverse ecological niches that harbor distinct microbiomes, and their organization is determined by genetic, metabolic and ecological factors. Over the last 10 years, significant progress has been made in understanding the role of genetic and metabolic drivers that influence plant beneficial microbiomes; however, ecological drivers remain mostly unexplored. There is a need to study the trio relationships among hosts, beneficial microbiomes and their ecological traits, which can provide incredible information about the core microbiome and its taxonomic and functional attributes. In this regard, integrating molecular biology, synthetic biology and ecology can be crucial for uncovering the complexity of the plant beneficial microbiome and its usage in the development of high-yielding, smart and climate-resilient crops in the future.

In this review, we provide a multiscale overview on the role of the plant beneficial microbiome in disease resistance, which has recently become one of the most exciting research in the field of plant stress biology. Below, we highlight a number of outstanding questions that need to be addressed in order to explore the potential of the plant beneficial microbiome in disease resistance. How does the plant beneficial microbiome mimic or evade the plant immune system? Does it have a similar approach of evading, or is it different from pathogens? How do plant immune system signatures, such as SA, JA and ET, as well as the trio crosstalk, influence beneficial microbiome assembly? How does the beneficial microbiome function against biotrophic and necrotrophic pathogens, and how does their signaling affect microbiome structure? How does the plant beneficial microbiome offer disease protection under multiple stresses [84]? Our understanding of the plant immune system is largely based on decades of research on the interactions between plants and pathogens. In the context of microbiome, this knowledge is currently being reviewed, evaluated and organized, finding fascinating contrasts and similarities [85]. Therefore, future research should focus on how the plant immune system reacts with different plant beneficial microbiome and on how it influences the particular beneficial microbiota that promote crop fitness and productivity. 

## Figures and Tables

**Table 1:** Shows the role of plant beneficial microbiome in plant disease resistance.

Host	Pathogens	Pathogen- or Host-Triggered Modulation of Beneficial Microbiome to Provide Host Resistance	Mechanism of Host Disease Resistance	References
*A. thaliana*	*Pseudomonas syringae* pv. tomato	Recruitment of Bacillus sps to suppress *Pseudomonas syringae*	Activation of ISR biofilm formation and chemotaxis	[42]
*Linum usitatissimum*	*F. oxysporum*	Enrichment of beneficial microbiome to suppress wilt disease	Production of antifungal metabolites, siderophores and cyanides	[43]
*Beta vulgaris*	*Rhizoctonia solani*	Enrichment of beneficial microbiota against *R. solnai*	Competition and production of antimicrobial compounds such as phenazine and chitinase	[28]
*B. vulgaris*	*R. solani*	Modification of the plant microbiome and enrichment of Oxalbacteraceae, Sphingobacteraceae, Burkholderiaceae, Sphingomonadaceae	Biofilm formation and the production of antifungal secondary metabolites	[44]
*Solanum lycopersicum*	*Fussarium oxysporum* f. sp. lycopersici	Modification of the plant microbiome and enrichment of Proteobacteria, Actinobacteria and Firmicutes, which protect against vascular wilt pathogens of the tomato	Inducing plant defenses against pathogens, ISR activation and production of antimicrobial compounds, cyclic lipopeptides and polyketides	[45]
Citrus	*Candidatus liberibacter*	Modification of the plant microbiome	ISR activation and the expression of plant defense-related genes	[46]
*Pinus roxburghii*	*Bursaphelenchus xylophilus*	Enrichment of endophytes	Production of antimicrobial compounds	[47]
*A. thaliana*	*Hyaloperonospora arabidopsis*	Enrichment of the beneficial microbiome	Activation of ISR biofilm formation and the production of antimicrobial agents	[34]
*S. lycopersicum*	*Botyritis cinerea*	Enrichment of *Trichoderma harzianum*	Biocontrol effect and rhizosphere competence	[48]
*S. lycopersicum*	*R. solanacearum*	Modification of the plant microbiome and enrichment of beneficial taxa	Biocontrol effect and rhizosphere competence	[31]
*A. thaliana*	*P. syringae* pv. Tomatao	Enrichment of the beneficial microbiome	Plant systemic signaling	[36]
*Cucumis sativus*	*F. oxysporum f.spfrr*	Enrichment of beneficial taxa, Comamonadaceae and Xanthomonadaceae	Antimicrobial chemicals and induced disease resistance	[36]
*Oryzae staiva*	*Xanthomonas oryzae*	Enrichment of endophytes	Production of biocontrol agents	[49]
*Fragaria × ananassa*	*Macrophomina phaseolina*	Modification of the plant microbiome and enrichment of beneficial taxa	Production of biocontrol agents	[50]

## Data Availability

Not applicable.
